# Supplementary material for: Circulating cell type senescence signatures track distinct dimensions of health status and trajectories in human longitudinal cohorts
Source: Cell Rep. Author manuscript; Available in PMC 2026 Jul 13. (PMC13358927; doi:10.1016/j.celrep.2026.117389)
Supplement: 1 [file NIHMS2190661-supplement-1.pdf]

Cell Reports, Volume *45*

Supplemental information

Circulating cell type senescence signatures track distinct dimensions of health status and trajectories in human longitudinal cohorts

Bradley Olinger, Carlos Anerillas, Allison B. Herman, Dimitrios Tsitsipatis, Reema Banarjee, Toshiko Tanaka, Julian Candia, Manolis Maragkakis, Stefania Bandinelli, Keenan A. Walker, Eleanor M. Simonsick, Yue A. Qi, Luigi Ferrucci, Myriam Gorospe, and Nathan Basisty

Supplemental Figure 1: Comparing Senescence and Non-Senescence Signatures for Trait Prediction

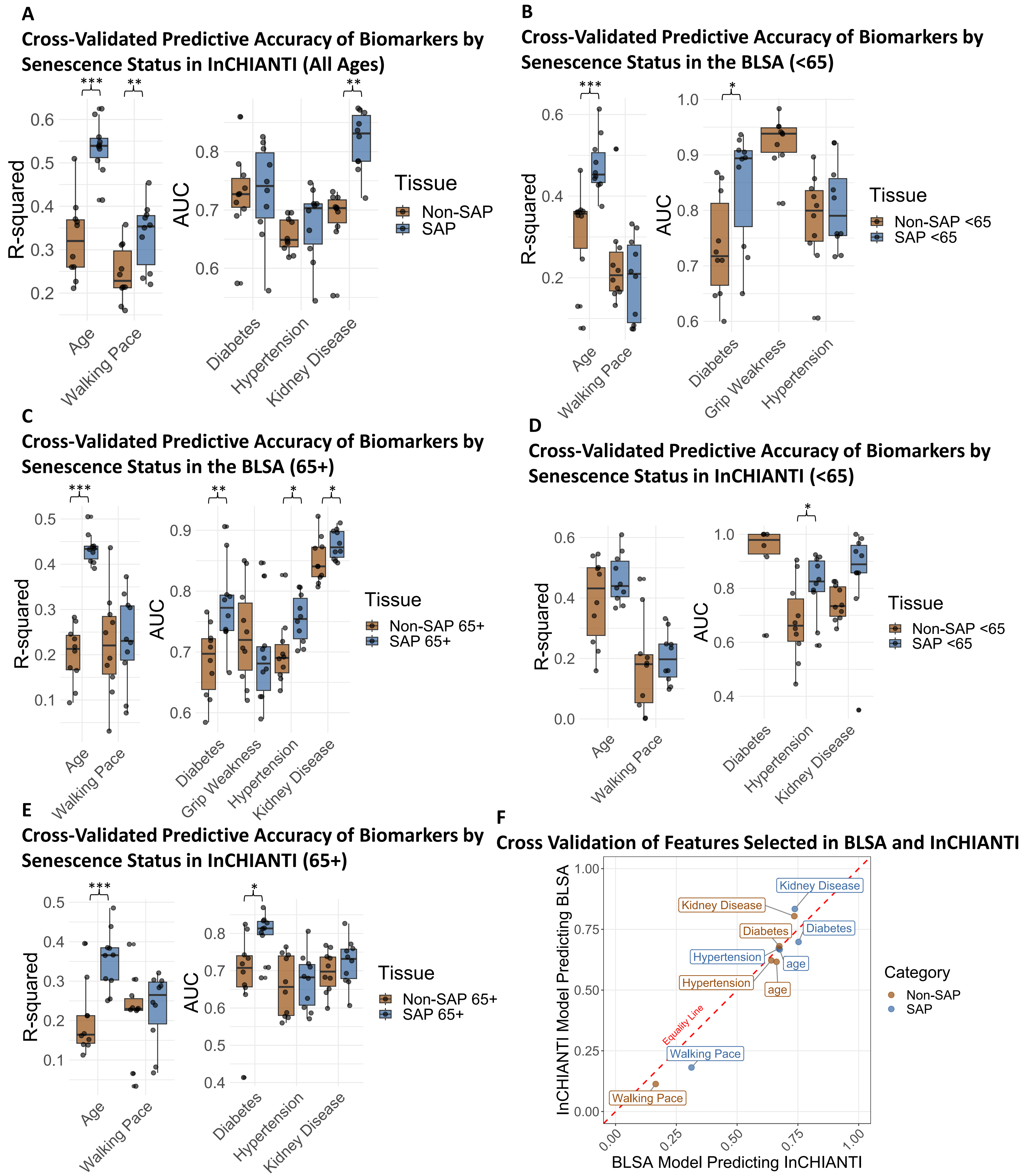

Supp. Figure 1: Cross-validated R-Squared values across ten iterations (90% train, 10% test) for clinical parameters. To normalize different pool sizes, only the top 25 ENSPs were used for each group of proteins, which were condensed into a mean composite score. **A)** SAP and Non-SAP senescence burden score trait prediction in the InCHIANTI. **B)** SAP and Non-SAP senescence burden score trait prediction in the BLSA among participants younger than 65. **C)** SAP and Non-SAP senescence burden score trait prediction in the BLSA among participants 65 years of age or older. **D)** SAP and Non-SAP senescence burden score trait prediction in InCHIANTI among participants younger than 65. **E)** SAP and Non-SAP senescence burden score trait prediction in the InCHIANTI among participants 65 years of age or older. *For groups younger than 65, the disease prevalence was low for conditions such as grip weakness, kidney disease, and diabetes, and so missing predictions reflect instances where no features were selected during elastic net modeling and thus model accuracy could not be assessed. Paired Student's T-Tests were used to compare SAP vs. Non-SAP parameter prediction. P-value significance: * <0.05 ** <0.005 *** <0.0005.

Supplemental Figure 2: Stratified Random Sampling of the BLSA and Ontology of Proteins Upregulated in Later Life

A
Age Distribution of Total BLSA Cohort, Eligible Participants (Ages 50-90), and Those Randomly Selected

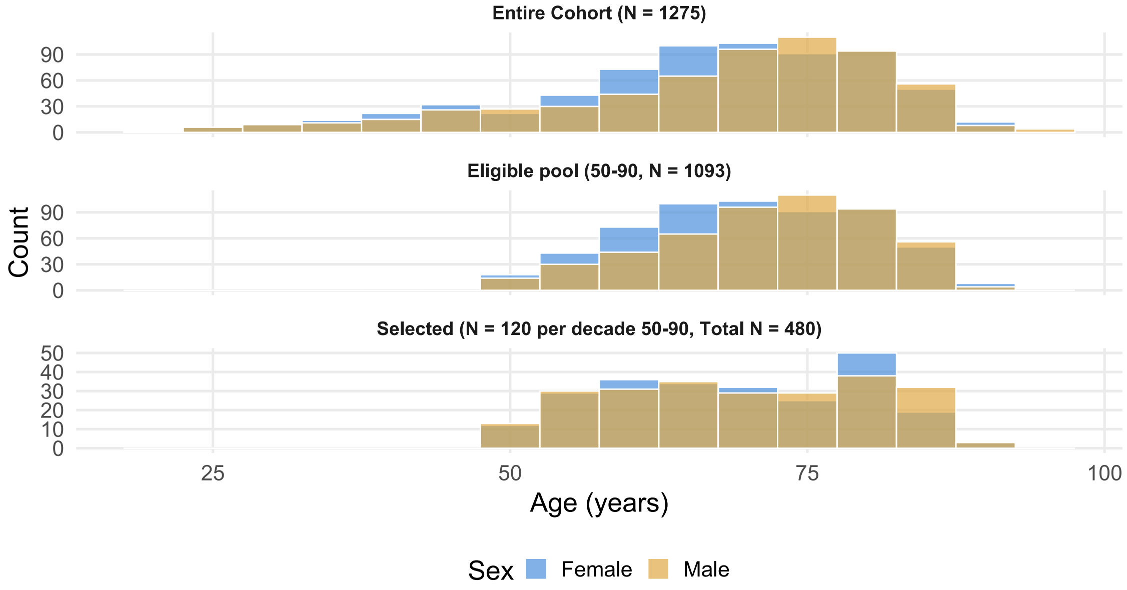

B
Overrepresentation Analysis of SAPs by Stage of Life

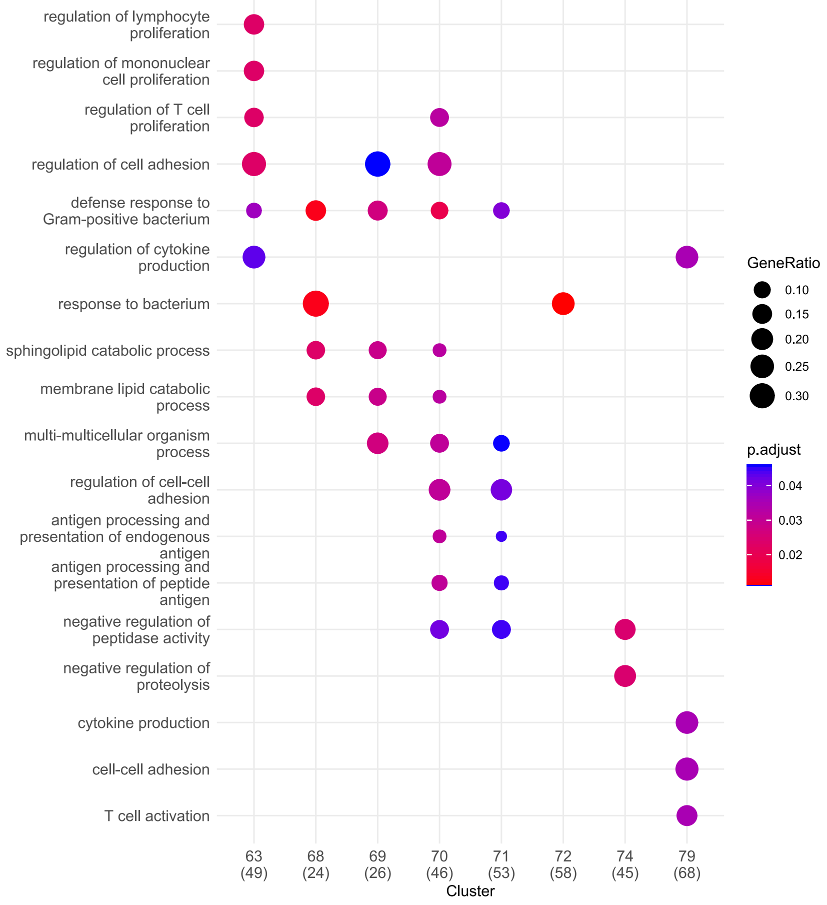

C
Overrepresentation Analysis of SAPs by Sex

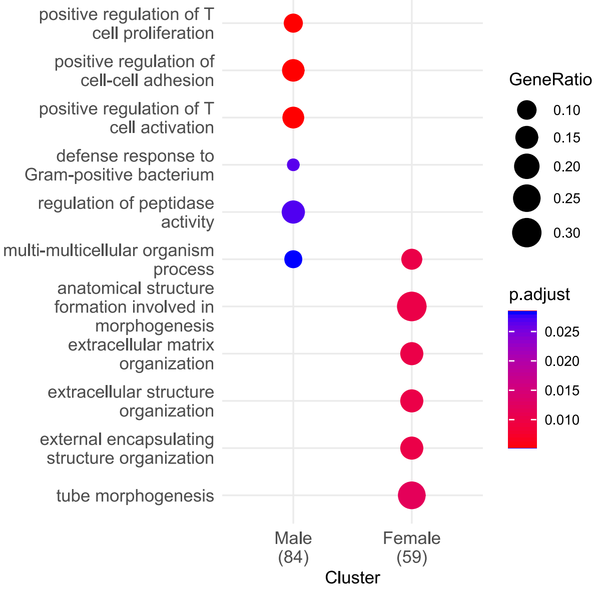

Supp. Figure 2: Differential Expression Sliding Window Analysis (DE-SWAN) was performed to identify SAPS and Non-SAPs upregulated by year from ages 60-80. **A)** Age distribution of the BLSA entire cohort, the eligible pool of individuals ages 50-90, and those randomly selected, N = 120 per decade, 50% male and 50% female. **B)** Overrepresentation analysis (Biological Process) of SAPs that are elevated by year according to the DE-SWAN method in the BLSA. **C)** Overrepresentation analysis (Biological Process) of SAPs that are elevated in males or females during the 60s or 70s according to the DE-SWAN method in the BLSA.

Supplemental Figure 3: Senescence Markers Identified by Tissue and Induction Method

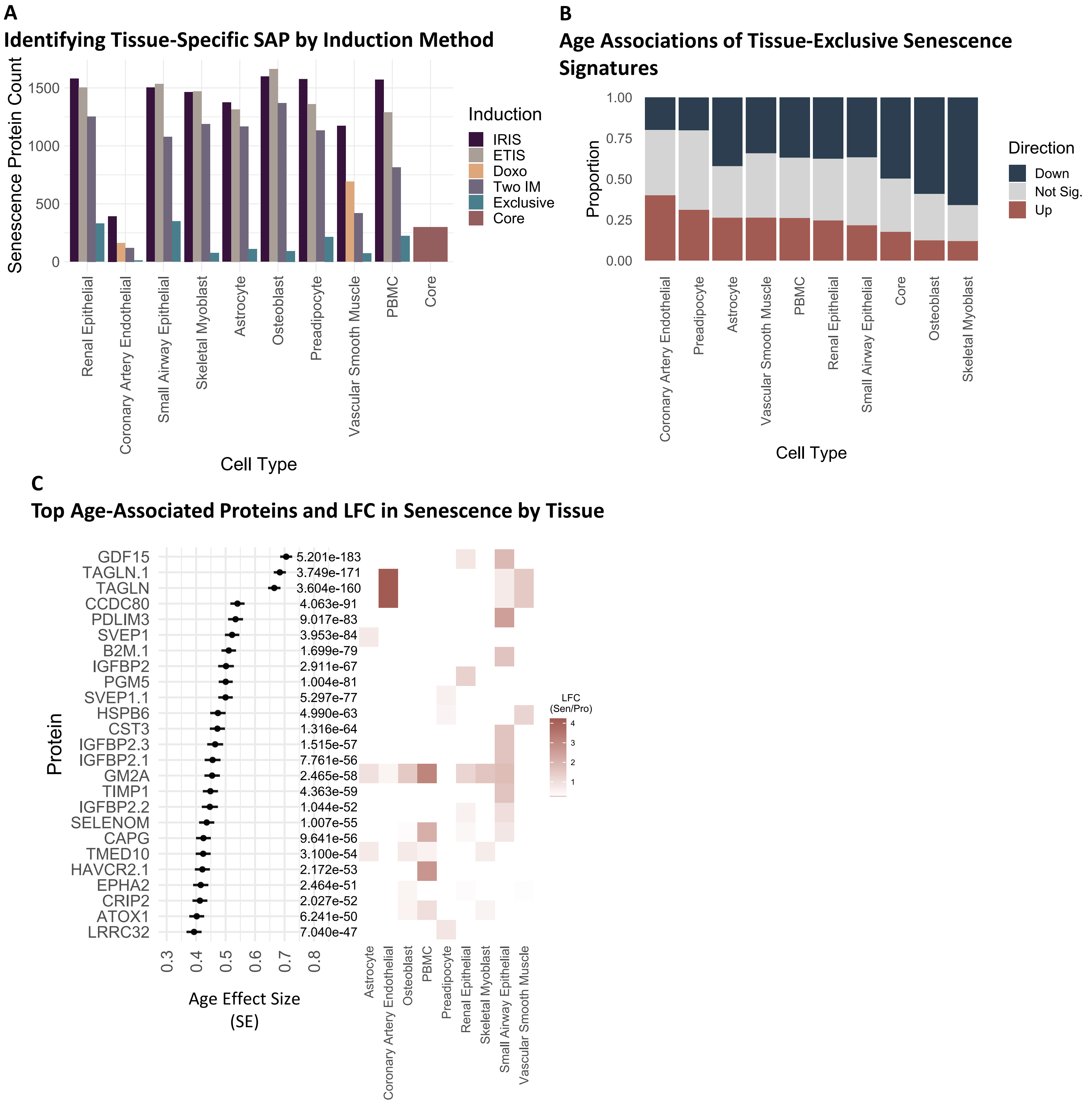

Supp. Figure 3: A) Senescence associated proteins (SAPs) were identified across cell types with multiple induction methods, including ionizing radiation (IRIS), etoposide (ETIS), and doxorubicin (DOXO). SAPs present in two induction methods (Two IM), as well as those present in two induction methods and exclusive to each cell type (Exclusive), were also identified by cell type. Last, a group of proteins that were elevated in two induction methods in at least 6 cell types were identified as a core senescence signature (Core). B) Linear modeling of cell type-exclusive SAPs revealed the proportion of each that were associated with age in the BLSA using the formula: age ~ protein + sex + race. C) The top 25 age-associated SAPs among all cell types in the SenCat by their effect size (+/- SE) and adjusted p-value for age, and their respective average log fold change (Sen/Pro) in each tissue.

*For Sen/Pro log fold change, only proteins significantly upregulated in that cell type using two induction methods are shown by their average LFC across both induction methods.

Supplemental Figure 4: Orthogonal Validation of Cell Type SAPs in the Human Protein Atlas

A

Orthogonal Validation of the SenCat Cell Type Senescence Signatures in the Human Protein Atlas

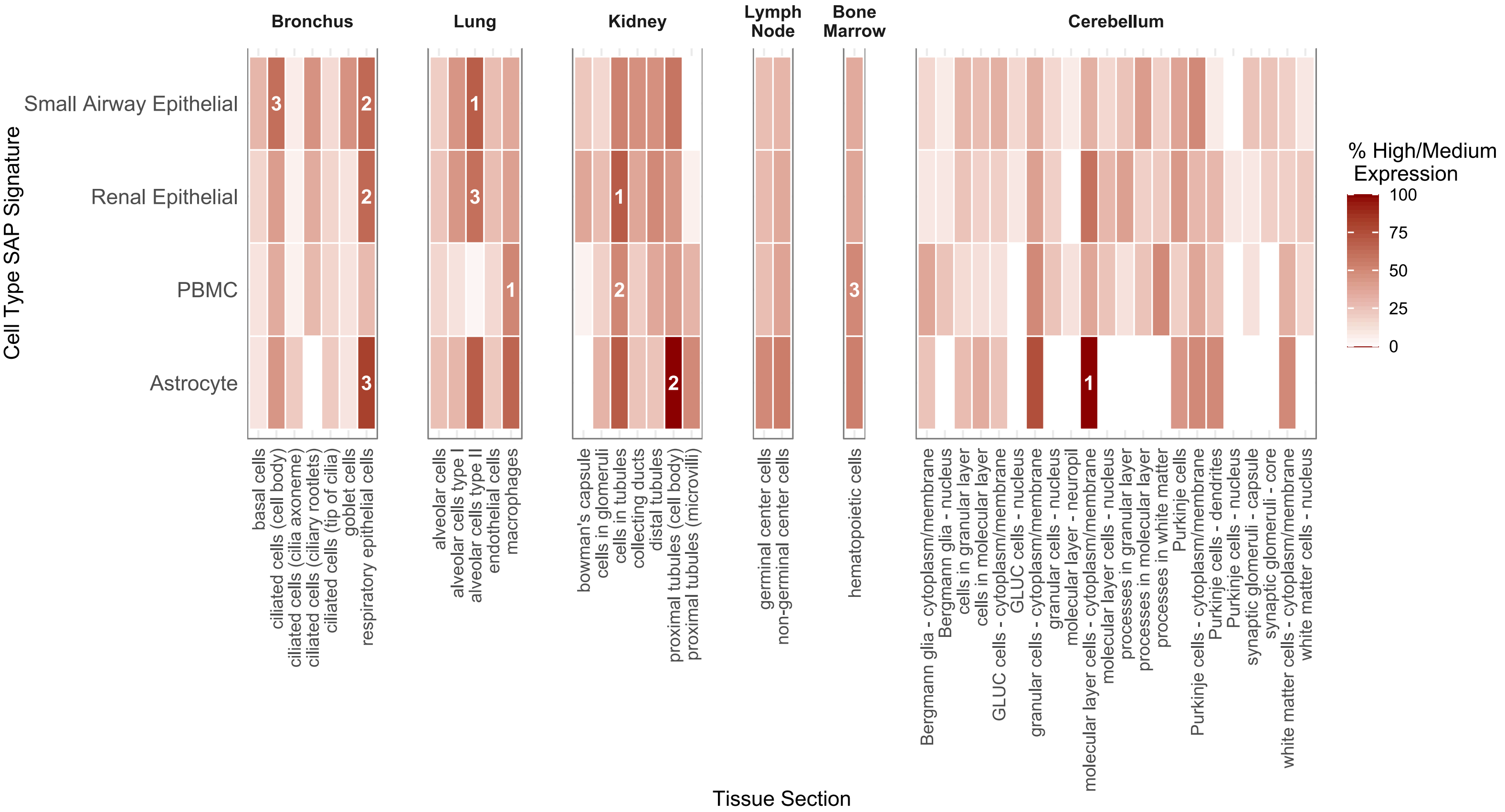

Supp. Figure 4: A) Four cell type enriched senescence signatures, including from small airway epithelial cells, renal epithelial cells, PBMCs, and astrocytes, were examined in the Human Protein Atlas in tissues that roughly correspond to the same organ system. The percent of the cell type SAPs for each signature with medium or high expression compared with no or low expression is shown. Numbers 1, 2, and 3 represent the three tissue sections where the highest percentage of medium or highly expressed proteins from each given cell type senescence signature was observed.

Supplemental Figure 5: Senescence Burden Associations with Clinical Parameters

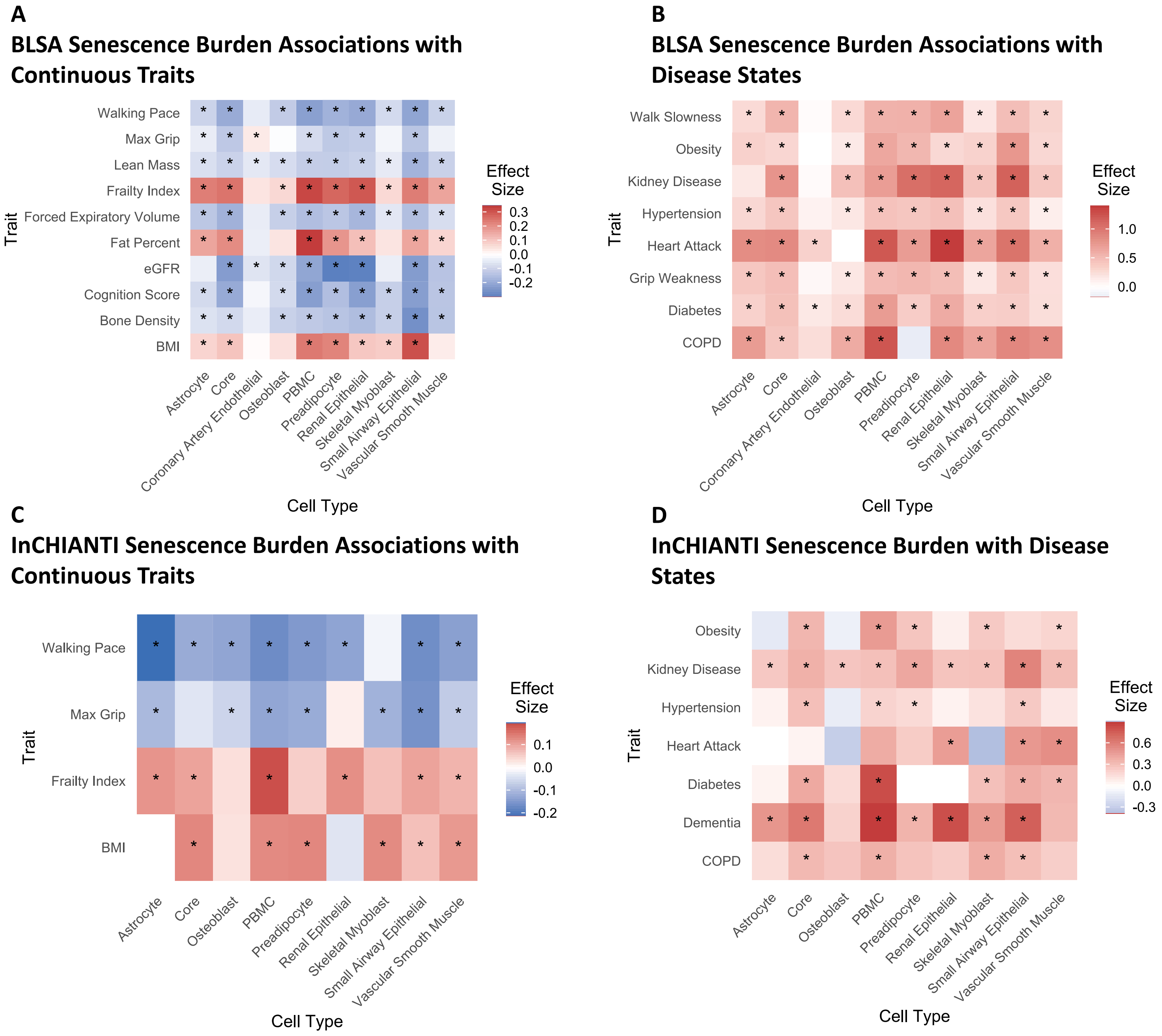

Supp. Figure 5: Up to 25 cell type-exclusive SAPs were selected using elastic net modeling for each cell type and clinical parameter combination. Each set was compiled into a mean composite score to represent cell type senescence burden implicated in clinical parameters. A) Unbiased display of cell type composite senescence burden associations via linear modeling with continuous clinical traits in the BLSA. B) Cell type composite Senescence Burden associations via logistic regression with disease status in the BLSA. C) Cell type composite Senescence Burden associations via linear modeling with continuous clinical traits in InCHIANTI. D) Cell type composite Senescence Burden associations via logistic regression with disease status in InCHIANTI. P-value significance: * <0.05

Supplemental Figure 6: Comparing Senescence and Non-Senescence Signatures for Trait Prediction

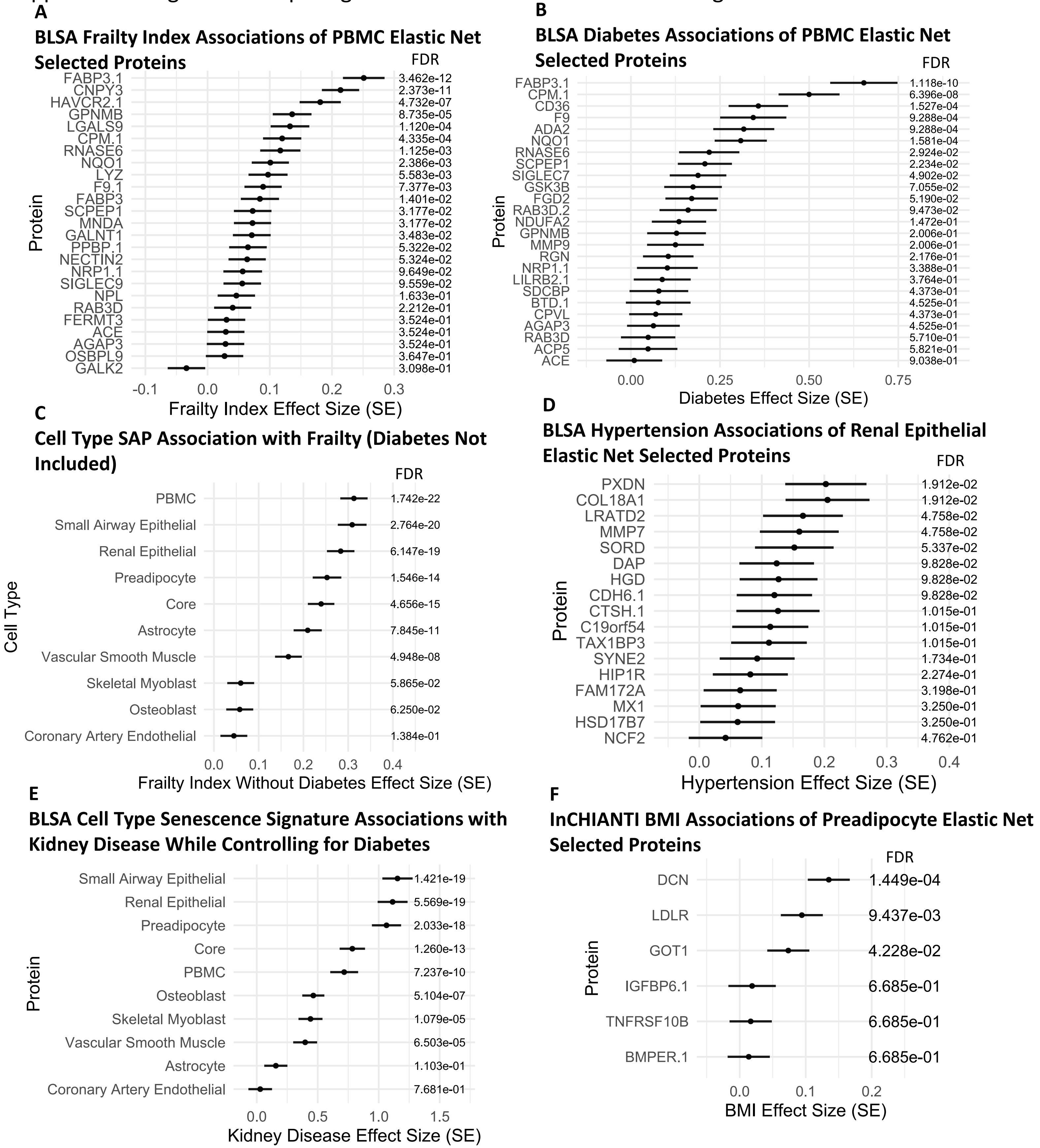

Supplemental Figure 6: Elastic Net Selected Proteins Associate with Frailty and Diabetes. Up to 25 elastic net selected SAPs were selected from cell type-unique senescence signatures as implicated in a frailty index or separately for diabetes status, hypertension, kidney disease, or BMI which were each compiled into a mean composite score to represent cell type-unique senescence burden. A) Frailty index effect size (+/- SE) via linear modeling for PBMC SAPs selected via elastic net modeling in the BLSA using the formula: frailty index ~ protein + age + sex + race. B) Diabetes effect size (+/- SE) via logistic regression for PBMC SAPs selected via elastic net modeling in the BLSA using the formula: diabetes ~ protein + age + sex + race. C) Cell Type SAP composite score effect size (+/- SE) with a 43-component Frailty score (Diabetes Excluded). D) Hypertension status effect size (+/- SE) with renal epithelial SAPs selected via elastic net modeling in the BLSA using logistic regression with the formula: hypertension status ~ protein + age + sex + race. E) Cell Type SAP composite score effect size (+/- SE) with kidney disease (Diabetes as Covariate). F) BMI effect size (+/- SE) via linear modeling for preadipocyte SAPS selected via elastic net modeling in InCHIANTI using the formula: BMI ~ protein + age + sex.

Supplemental Figure 7: Tissue-Unique Senescence Signatures Show Unique Clinical Predictive Capacity

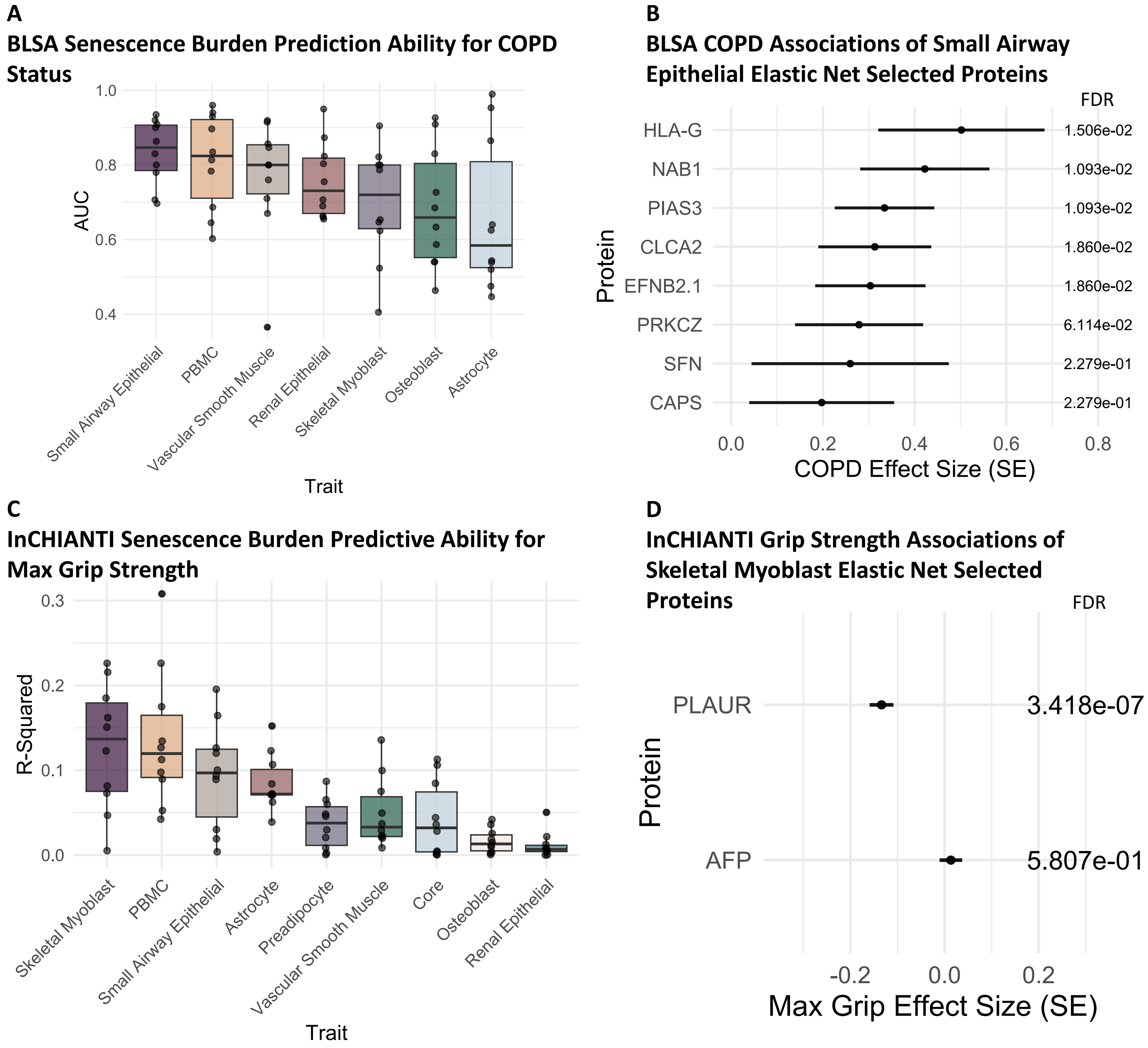

Supp. Figure 7: Up to 25 cell type-exclusive SAPs were selected via elastic net modeling across cell types and were each compiled into a mean composite score to represent cell type senescence burden implicated in clinical parameters. A) The cross-validated predictive ability of cell type senescence burden and COPD in the BLSA. B) COPD effect size (+/- SE) via logistic regression for Small Airway Epithelial SAPs selected via elastic net modeling in the BLSA. C) The cross-validated predictive ability of cell type senescence burden and Max Grip Strength in InCHIANTI. D) Max Grip Strength effect size (+/- SE) via linear modeling for Skeletal Myoblast SAPs selected via elastic net modeling in InCHIANTI.

Supplemental Figure 8: Tissue-Specific Senescence Burden Reveals Longitudinal Health Trajectories

A
PBMC Senescence Signatures Show the Strongest Associations with Kidney Disease Onset in the BLSA

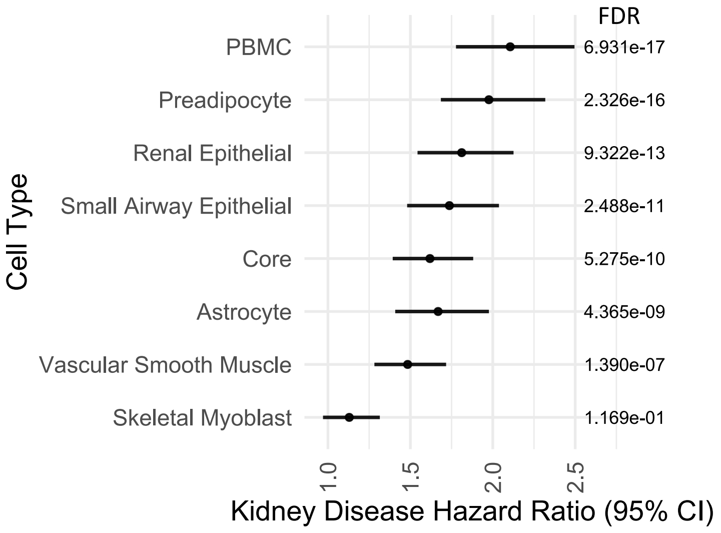

B
PBMC Senescence Signatures Show the Strongest Associations with Hypertension Onset in the BLSA

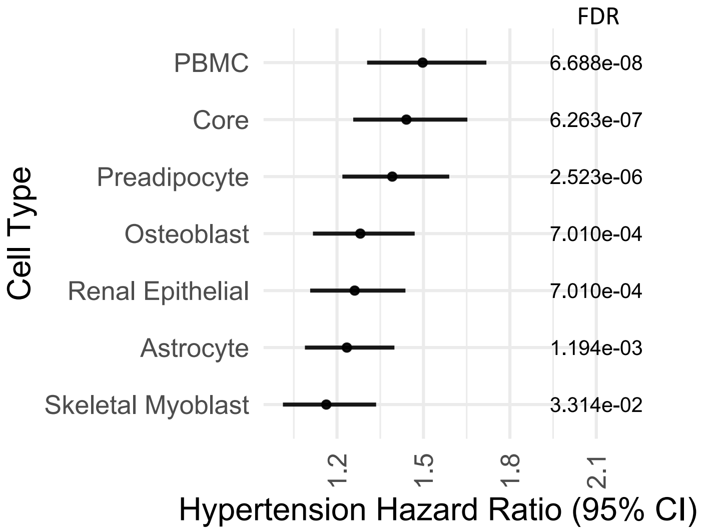

Supp. Figure 8: Up to 25 cell type-exclusive SAPS were selected via elastic net modeling and each set was compiled into a mean composite score to represent cell type senescence burden across multiple cell types. **A**) The hazard ratio (95% CI) between the cell type senescence burden and kidney disease onset via cox proportional hazards modeling in the BLSA. **B**) The hazard ratio (95% CI) of the cell type senescence burden and hypertension onset via cox proportional hazards modeling in the BLSA.

Supplemental Figure 9: Tissue-Specific Seno-Age Shows Different Trajectories

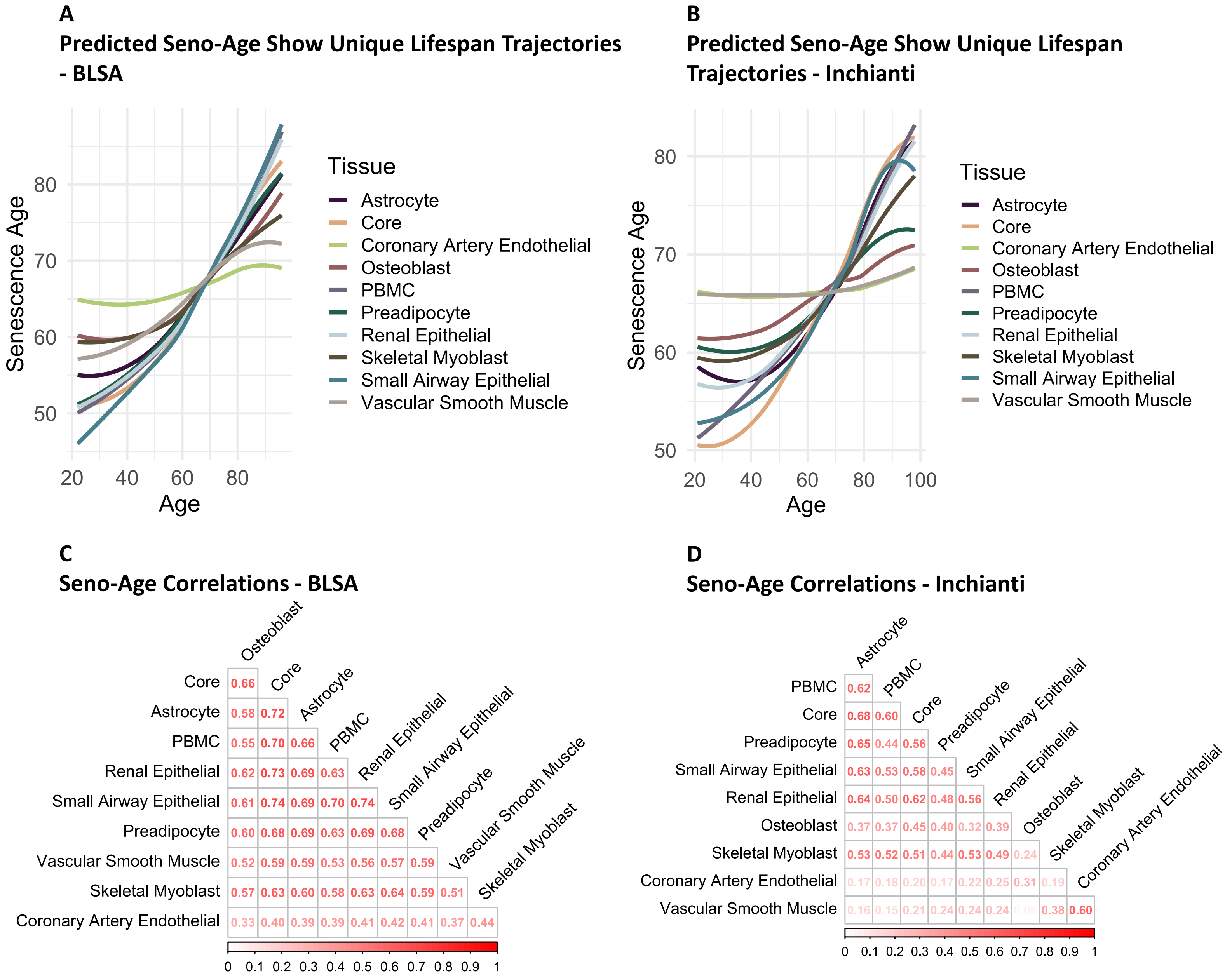

Supp. Figure 9: A) Up to 25 cell type-exclusive SAPs were selected via elastic net modeling to predict age for each cell type. These ENSPs were used to model age using linear modeling for each SenCat cell type, termed Seno-Age. Seno-age trajectories are shown by cell type and chronological age in the BLSA. B) As in A, seno-age is shown by cell type and chronological age in InCHIANTI. C) Seno-Age spearman correlations are shown by cell type in the BLSA D) As in C, Seno-Age spearman correlations are shown by cell type in Inchianti.

Supplemental Figure 10: Deriving Senescence Age and Senescence Age Gaps in the BLSA

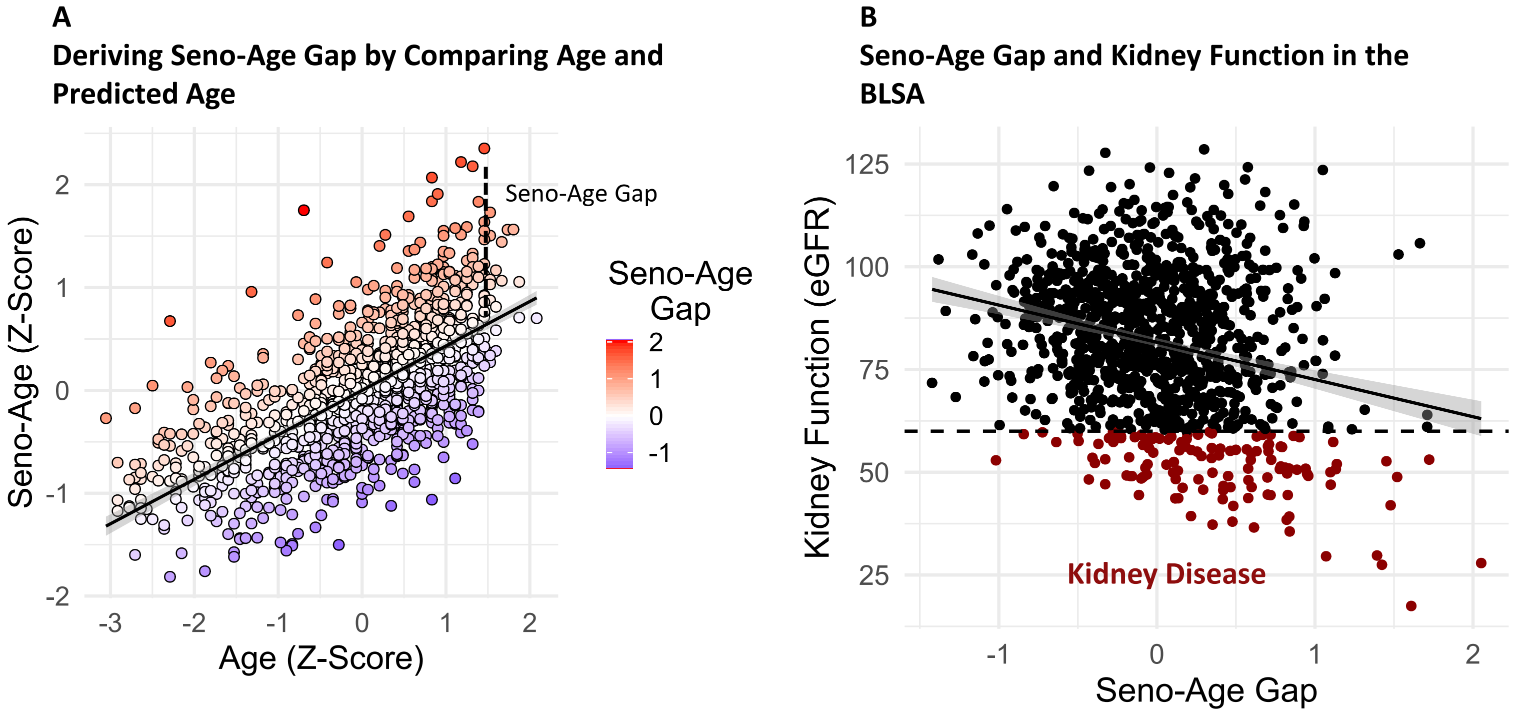

Supp. Figure 10: Up to 25 cell-type exclusive SAPs were selected using elastic modeling for each cell type. Each set was used to predict age using a linear model for each individual, representing "seno-age" for each cell type. Seno-age gaps were created using the residual of a linear trend line between chronological age and modeled seno-age. **A**) Example seno-age shown is predicted using astrocyte elastic net selected SAPs and compared with chronological age. **B**) Example of seno-age gap compared with kidney function in the BLSA. The seno-age gap shown was determined using astrocyte elastic net selected SAPs.

Supplemental Figure 11: Seno-Age Gap Associations with Clinical Parameters

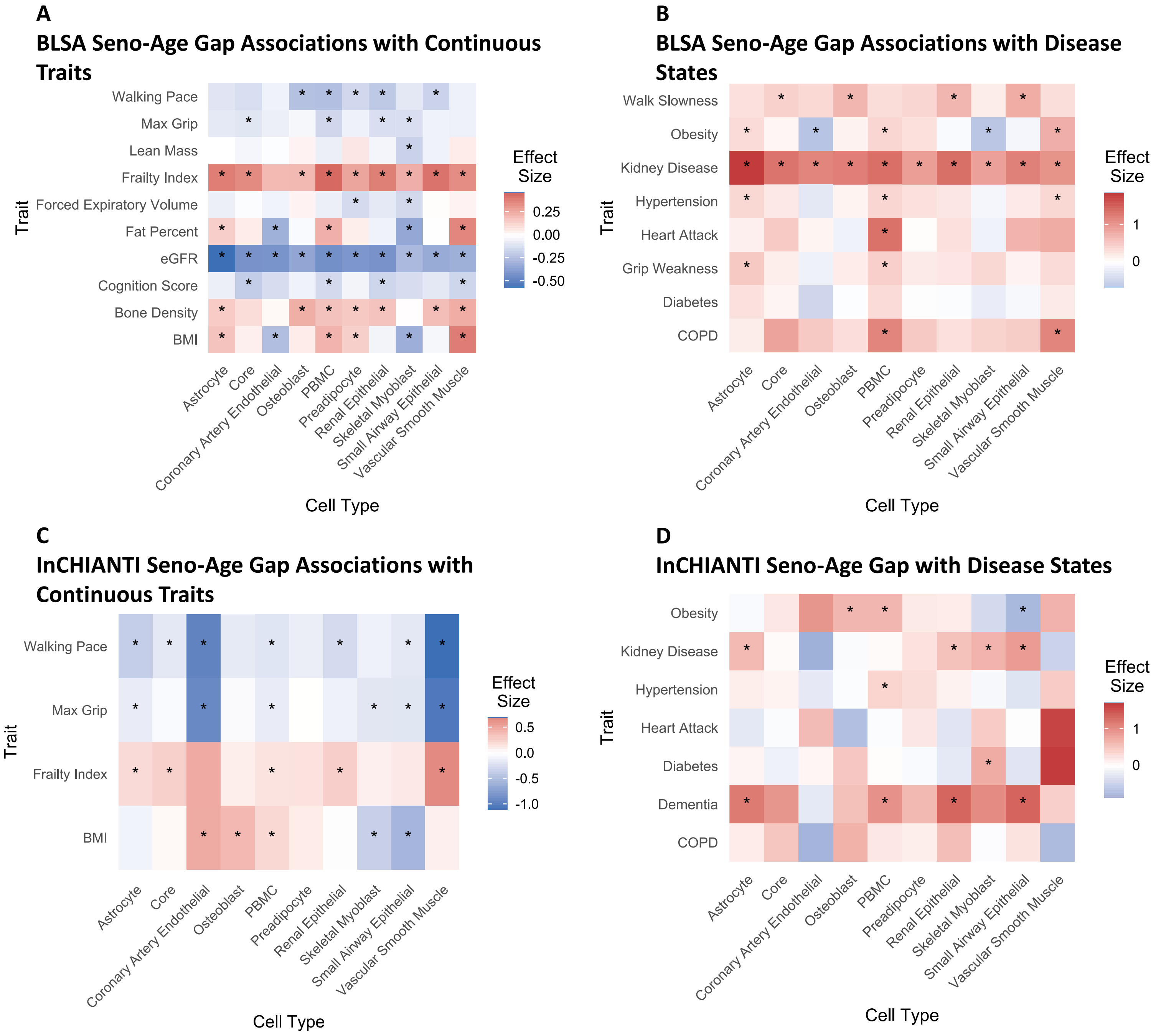

Supp. Figure 11: Up to 25 cell type-exclusive SAPs selected via elastic net modeling were used to model age within a linear model across multiple cell types, termed cell type seno-age. From here, cell type seno-age gaps were produced using the residuals of a trend line between seno-age and chronological age. A) Unbiased display of Seno-Age Gap associations via linear modeling with continuous clinical parameters in the BLSA. B) Seno-age gap associations via logistic regression with disease status in the BLSA. C) Seno-Age Gap associations via linear modeling with continuous clinical parameters in InCHIANTI. D) Seno-Age Gap associations via logistic regression with disease status in InCHIANTI. P-value significance: * <0.05.

Supplemental Table 1: Senescence induction dose by cell type.

Cell Type	Senescence trigger	Treatment details	Days into senescence
HCAEC (Arterial Endothelial)	Doxo	250 nM, 3 days	8
HCAEC (Arterial Endothelial)	IR	15 Gy	8
HUVEC (Venal Endothelial)	Etoposide	10 uM, 3 days	8
HUVEC (Venal Endothelial)	IR	7.5 Gy	8
HSAEC (Lung Epithelial)	Etoposide	20 uM, 3 days	8
HSAEC (Lung Epithelial)	IR	10 Gy	8
BJ (Skin Fibroblasts)	Etoposide	25 uM, 6 days (refreshed at day 3)	8
BJ (Skin Fibroblasts)	IR	15 Gy	8
WI-38 (Lung Fibroblasts)	Etoposide	50 uM, 6 days (refreshed at day 3)	8
WI-38 (Lung Fibroblasts)	IR	15 Gy	8
HSKM (Myoblasts)	IR	10 Gy	8
HSKM (Myoblasts)	Etoposide	20 uM	8
PreAdipo (Subcutaneous Preadipocytes)	IR	15 Gy	8
PreAdipo (Subcutaneous Preadipocytes)	Etoposide	25 uM	8
HVSMC (Vascular Smooth Muscle)	Doxo	85 nM, 3 days	8
HVSMC (Vascular Smooth Muscle)	IR	15 Gy	8
HEKn (Skin Keratinocytes)	Etoposide	25 uM, 3 days	8
HEKn (Skin Keratinocytes)	IR	10 Gy	8
HEMn (Melanocytes)	Etoposide	25 uM, 3 days	8
HEMn (Melanocytes)	IR	7.5 Gy	8
NHO (Osteoblasts)	Etoposide	20 uM, 3 days	8
NHO (Osteoblasts)	IR	10Gy	8
NHA (Astrocytes)	Etoposide	20 uM, 3 days	8
NHA (Astrocytes)	IR	10Gy	8
PBMC (Peripheral Blood Mononuclear)	Etoposide	20 uM, 3 days	6
PBMC (Peripheral Blood Mononuclear)	IR	7.5 Gy	6
HREC (Renal Mixed)	Etoposide	20 uM, 3 days	8
HREC (Renal Mixed)	IR	10 Gy	8

Supplemental Table 2: Clinical Parameters in the BLSA - Data reported as mean (standard deviation).

	All	20 to 40	40 to 60	60 to 70	80+	N
Count	1275	70	252	702	251	
Age	67 (14.1)	32.5 (4.6)	51.2 (6)	70.2 (5.4)	83.4 (3.2)	
Female Percent	52.8	54.3	56.3	52.3	50.2	
BMI	26.9 (4.6)	25.1 (4.8)	27.1 (4.8)	27.5 (4.8)	25.6 (3.3)	1269
Walking Pace	1.8 (0.4)	2.1 (0.3)	2 (0.3)	1.8 (0.3)	1.6 (0.3)	983
eGFR	81.6 (17.6)	107.2 (12.6)	93.3 (13.6)	77.9 (15.1)	69.8 (13.6)	1120
Bone Density	1.1 (0.2)	1.1 (0.1)	1.1 (0.2)	1.1 (0.2)	1.1 (0.2)	1022
Lean Mass	7.5 (1.3)	7.9 (1.3)	7.9 (1.4)	7.4 (1.3)	7.1 (1.1)	1021
Fat Percent	35.3 (9.4)	29.5 (10.1)	34.3 (9.3)	37 (9.2)	33.6 (8.5)	1021
Forced Expiratory Volume	2.5 (0.9)	3.5 (0.9)	3 (0.9)	2.4 (0.8)	2 (0.7)	1001
Max Grip	33.5 (11.4)	40.7 (12)	38.9 (11.7)	32.2 (10.1)	26.8 (9.2)	1019
Frailty Index	0.1 (0.1)	0 (0)	0.1 (0)	0.1 (0.1)	0.1 (0.1)	980
Cognition Score	28.5 (1.6)	29.3 (0.9)	29.2 (1)	28.5 (1.7)	28.1 (1.7)	1047

Supplemental Table 3: Clinical Parameters in InCHIANTI - Data reported as mean (standard deviation).

	All	20 to 40	40 to 60	60 to 70	80+	N
Count	997	99	104	665	129	
Age	66.3 (15.4)	30.3 (5.2)	49.5 (5.8)	70.7 (4.5)	84.9 (3.6)	
Female Percent	55	54.5	49	54.3	63.6	
BMI	27.2 (4.1)	24.3 (3.6)	27.2 (4)	27.8 (4.1)	26.1 (3.9)	962
Walking Pace	1.5 (0.4)	1.9 (0.3)	1.8 (0.3)	1.5 (0.3)	1.1 (0.4)	934
Max Grip	33.1 (13.8)	46.2 (13.5)	43.3 (14.4)	31.7 (12)	21.8 (8.5)	843
Frailty Index	1.6 (0.8)	NaN (NA)	NaN (NA)	1.5 (0.7)	1.9 (0.9)	334

Supplemental Table 4: Counts and Proportions of SAP and Non-SAP in the BLSA and InCHIANTI Studies

Raw Numbers			
	SAP	Non-SAP	Inconclusive
BLSA	1941	1266	737
InCHIANTI	355	228	129
Fisher's Exact Test P-Value: 0.9332			
Proportions			
	SAP	Non-SAP	Inconclusive
BLSA	49.21399594	32.09939148	18.68661258
InCHIANTI	49.85955056	32.02247191	18.11797753

Supplemental Table 8: Features Selected in Both BLSA and InCHIANTI Studies for Predicting Traits

Trait	BLSA	InCHIANTI	ModelType	Type	Features
age	0.685414306	0.6729173	Linear	SAP	Q99988/Q76M96/P00750/Q9GZN4/Q9UBP4.1/Q12841/P24593.1/P46531/Q9UM47/O15031.1/Q12860/P52799/P40121/P00747.2/Q8N423/P22897/P35442.1/P05155.1/P17931/P09758.1/Q99538/P35613/P45985/P15260/Q03154/Q10588/O95881.1/P08758/P28799/P42892/P20936
Walking Pace	0.311743502	0.180708363	Linear	SAP	P05413.1/Q76M96/P09237/P17931/Q01469/Q99988/Q13813
Hypertension	0.669854722	0.671115417	Logistic	SAP	P05413.1/Q99988/O14786.1/P00734.1/P09237/Q8TDQ0.1/Q9Y286/Q10588/Q08380
Diabetes	0.75122549	0.69670351	Logistic	SAP	Q99988/O15031.1/O00764/P16671/P06396.1/P18065.2
Kidney Disease	0.73802823	0.834049077	Logistic	SAP	P61769.1/Q96HD1/Q01973.1/P39060
age	0.664525355	0.617492387	Linear	Non-SAP	Q99988/Q76M96/P00750/Q9GZN4/Q9UBP4.1/Q12841/P24593.1/P46531/Q9UM47/O15031.1/Q12860/P52799/P40121/P00747.2/Q8N423/P22897/P35442.1/P05155.1/P17931/P09758.1/Q99538/P35613/P45985/P15260/Q03154/Q10588/O95881.1/P08758/P28799/P42892/P20936
Walking Pace	0.16515581	0.112268806	Linear	Non-SAP	P05413.1/Q76M96/P09237/P17931/Q01469/Q99988/Q13813
Hypertension	0.640447942	0.622111567	Logistic	Non-SAP	P05413.1/Q99988/O14786.1/P00734.1/P09237/Q8TDQ0.1/Q9Y286/Q10588/Q08380
Diabetes	0.672690918	0.680749467	Logistic	Non-SAP	Q99988/O15031.1/O00764/P16671/P06396.1/P18065.2
Kidney Disease	0.734198832	0.803495021	Logistic	Non-SAP	P61769.1/Q96HD1/Q01973.1/P39060

Supplemental Table 9: Standardized Mean Differences between a group included in DE-SWAN and an excluded group.

Trait	Selected Group	Removed Group	SMD
Age	69.3 (10.7)	72.2 (7.9)	-0.312
Female	50.00%	44.50%	0.11
BMI	27.4 (4.6)	26.9 (4.5)	0.114
Walking Pace	1.8 (0.4)	1.8 (0.3)	0.087
Diabetes	14.20%	18.20%	-0.107
Hypertension	49.40%	48.90%	0.009

Supplemental Table 10: Validating Proportional Hazards Assumptiom of SAPs Implicated in Mortality

Protein	chisq	pval	symbol
P09104.1	0.094544687	0.758477132	ENO2
P55058	0.049958195	0.823136034	PLTP
O00391	1.990745366	0.158262967	QSOX1
P30085.1	1.325517206	0.24960428	CMPK1
Q9H1C4	0.211836516	0.645331754	UNC93B1
Q9UBI4	1.254626748	0.26267063	STOML1
Q9UKA8	4.186452811	0.040748273	RCAN3
Q8NFI3	0.033245735	0.855320516	ENGASE
P09237	7.60555959	0.00581886	MMP7
P42785	5.449954648	0.019568835	PRCP
Q13162	2.914742697	0.087773411	PRDX4
P35442.1	7.518723445	0.006106094	THBS2
P05120	1.160230622	0.281417717	SERPINB2
Q8NBJ7	6.888008952	0.008677587	SUMF2
